# Serum vitamin D levels and fall risk in adults: A cross-sectional study using the morse fall scale

**DOI:** 10.1371/journal.pone.0357028

**Published:** 2026-08-27

**Authors:** Chenliang Zhao, Jianrong Xiong, Yongxiang Li

**Affiliations:** 1 Department of Critical Care Medicine, Heyou Hospital, Foshan City, Guangdong, China; 2 Department of Rehabilitation, Xinhua Hospital, School of Medicine, Shanghai Jiao Tong University, Shanghai, China; 3 Department of Rehabilitation Medicine, The Fourth Affiliated Hospital Zhejiang University School of Medicine, Yiwu, Jinhua, Zhejiang, P.R. China; Universitas Muhammadiyah Aceh, INDONESIA

## Abstract

**Aims:**

This study aimed to examine the association between serum 25-hydroxyvitamin D [25(OH)D] concentrations and fall risk assessed using the Morse Fall Scale (MFS).

**Methods:**

This cross-sectional study included 131 hospitalized adults. Serum 25(OH)D concentrations were measured after admission, and fall risk was assessed using the MFS. Participants were classified as having low, moderate, or high fall risk. Multivariable logistic regression was used to evaluate factors associated with high fall risk.

**Results:**

Among 131 participants, 4, 15, and 112 were classified as having low, moderate, and high fall risk, respectively. Serum 25(OH)D concentrations differed across the three groups (p = 0.003) and were lowest in the high-risk group. In the adjusted model, each 1-nmol/L increase in serum 25(OH)D was associated with lower odds of high fall risk (adjusted OR = 0.93, 95% CI 0.89–0.96; p < 0.001), whereas older age was associated with higher odds (adjusted OR = 1.07 per year, 95% CI 1.02–1.13; p = 0.01). Sex was not independently associated with high fall risk (p = 0.79).

**Conclusions:**

Lower serum 25(OH)D concentrations and older age were independently associated with high MFS-defined fall risk in this hospitalized cross-sectional sample. Prospective studies are needed to determine temporality and whether correcting vitamin D deficiency reduces subsequent falls.

## Introduction

Falls among the elderly have an extremely high incidence and mortality rates. An article notes that “falls not only place a physiological and psychological burden on patients and families, but also on the healthcare system [1,2]. According to the Global Burden of Disease Study 2017, falls represent a leading cause of unintentional injury-related mortality worldwide [3]. Injuries from falling and falls would be known to affect the functional ability of older people living in the community and quality of life significantly [4]. Based on the results of these studies, it is imperative to focus on research to reduce the incidence and severity of falls in older people [2].

Vitamin D is a fat-soluble vitamin measured by the serum 25-hydroxyvitamin D 25(OH)D concentrations. According to earlier studies, the major functions of vitamin D are to regulate calcium and phosphorus levels and to support bones. Research shows that vitamin D receptor (VDR) is present in skeletal muscle [5] and that animal studies show that the lack of VDR in muscle cells of mice causes sarcopenia and lower muscle function [6]. Vitamin D can enhance balance and boost muscle strength [7,8].

Vitamin D replacement therapy positively affects balance and quality of life in postmenopausal women [9]. Researches have reasoned that vitamin D supplementation may reduce falls [10]; However, the efficacy of vitamin D supplementation in fall prevention remains a subject of ongoing debate. Recent large-scale clinical trials have indicated that such interventions may not yield significant benefits in populations that are already vitamin D-replete [11,12]. These findings suggest that the protective effect of vitamin D might be threshold-dependent, primarily manifesting in individuals with baseline deficiency rather than those with sufficient levels.

Despite biologic plausibility and extensive supplementation research, the association between measured serum 25(OH)D concentrations and clinically assessed fall risk remains insufficiently characterized in hospitalized rehabilitation populations. Prior trials have produced inconsistent results, often in populations with different baseline vitamin D status, and many studies have focused on incident falls rather than contemporaneous multidomain risk assessed using the MFS. We therefore examined whether serum 25(OH)D concentration was independently associated with MFS-defined high fall risk in hospitalized adults. Clarifying this association may help identify patients who warrant closer fall-risk assessment while providing a basis for prospective studies of temporality and intervention.

## Methods

### Study design and participants

**Fig 1:**
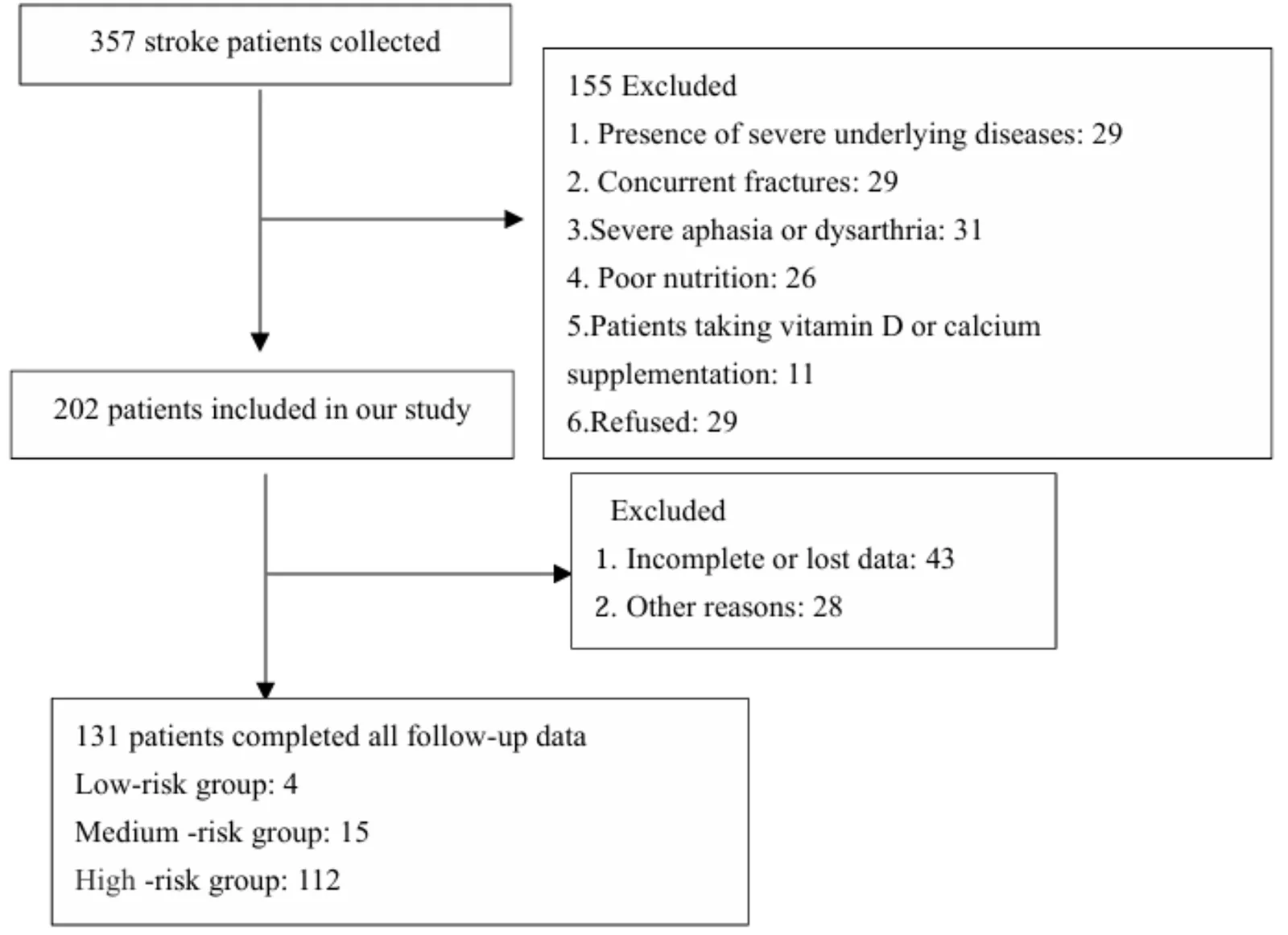
Flowchart of participant screening and enrollment. Flow diagram showing patient screening, exclusion criteria, and final sample size for this cross‑sectional study.

This study was approved by the Ethics Committee of the Fourth Affiliated Hospital, Zhejiang University School of Medicine (Approval No. 2025KE0801−2). Written informed consent was obtained from all participants after they received information about the study purpose, procedures, potential risks, and benefits. We screened 357 hospitalized patients whose records were collected from May 3, 2022, to December 13, 2022; the data were accessed for research purposes from August 10 to August 25, 2025. Inclusion criteria were the ability to participate in study procedures and provision of signed informed consent. At the initial screening stage, 155 patients were excluded because of severe underlying disease (n = 29), concurrent fracture (n = 29), severe aphasia or dysarthria that prevented assessment (n = 31), poor nutritional status (n = 26), current vitamin D or calcium supplementation (n = 11), or refusal to participate (n = 29). Of the remaining 202 patients, 43 were excluded because of incomplete or lost data and 28 for other prespecified eligibility reasons. Thus, 131 participants were included in the final cross-sectional analysis (Fig 1).

### Data collection

#### Clinical measurements.

A standardized questionnaire was used to collect demographic and clinical information, including alcohol consumption, sex, hypertension, diabetes, hyperlipidemia, body mass index (BMI), smoking history, and age. Fall risk was assessed using the MFS, with higher scores indicating greater risk. Participants were classified as low risk (0–24 points), moderate risk (25–44 points), or high risk (≥45 points) [13].

#### Laboratory tests.

Venous blood samples were collected on the morning of the second day after admission. Serum 25(OH)D, neuron-specific enolase (NSE), homocysteine (Hcy), C-reactive protein (CRP), fasting blood glucose (Glu), and platelet count (Plt) were measured. Vitamin D status was categorized using the prespecified thresholds of deficiency (<25 nmol/L), insufficiency (25 to <50 nmol/L), and sufficiency (≥50 nmol/L) [14]. Although some international guidance has used a higher sufficiency threshold (≥75 nmol/L) [15], the 50-nmol/L cutoff was applied consistently in this study.

### Statistical analysis

Categorical variables are presented as counts and percentages, and continuous variables as mean ± standard deviation or median (interquartile range), according to their distributions. Differences across the three MFS risk groups were evaluated using one-way analysis of variance for approximately normally distributed continuous variables or the Kruskal-Wallis H test for non-normally distributed continuous variables. Categorical variables were compared using the χ² test or Fisher’s exact test, as appropriate. Multivariable binary logistic regression was used to identify factors independently associated with high fall risk (high risk versus low/moderate risk), with serum 25(OH)D, age, sex, and BMI included as covariates. Results are reported as odds ratios (ORs) with 95% confidence intervals (CIs). A two-sided p value <0.05 was considered statistically significant.

## Results

### Characteristics of study participants

A total of 357 patients were screened. After 155 patients were excluded at the initial screening stage, 202 underwent further eligibility and data-quality assessment. Of these, 71 were excluded, leaving 131 participants in the final cross-sectional analysis (Fig 1). Based on MFS scores, 4 participants were classified as low risk, 15 as moderate risk, and 112 as high risk. Table 1 summarizes the demographic and clinical characteristics of the three groups. Serum 25(OH)D concentrations differed significantly across the groups (p = 0.003).

**Table 1:** Demographic and clinical characteristics of Morse fall risk.

Characteristics	*Morse*			*P*-value
	Low risk (n = 4)	Medium risk (n = 15)	High risk (n = 112)	
Baseline characteristics				
Age (y), mean ± SD	50.25 ± 13.15	51.13 ± 14.31	57.94 ± 13.44	0.12
Sex (female/male), n	0/4	3/12	35/77	**<0.001**
BMI, mean±SD	25.99 ± 4.00	25.27 ± 3.64	25.26 ± 3.29	0.91
Hypertension	1	8	79	0.07
Diabetes mellitus	1	1	26	0.31
Hyperlipidemia	0	2	17	1.00
Smoking history	3	6	36	0.20
Drinking alcohol	3	2	32	0.49
Vitamin D (nmol/l), median (IQR)	60.74 (45.27, 125.27)	53.35 (29.98, 61.85)	37.16 (26.91, 46.78)	**0.003**
CRP, median (IQR)	1.40 (0.31, 3.45)	1.30 (0.50, 2.30)	1.65 (0.70, 3.60)	0.43
NSE, median (IQR)	12.69 (10.04, 15.95)	10.75 (9.37, 13.60)	12.52 (9.89, 15.24)	0.50
Glu, median (IQR)	5.93 (4.57, 9.23)	4.70 (4.31, 5.23)	5.16 (4.49, 6.31)	0.25
Plt	233.00 ± 38.76	244.47 ± 34.52	266.17 ± 65.83	0.51
Hcy, median (IQR)	26.85 (5.25, 57.60)	13.60 (10.30, 17.90)	12.50 (10.60, 23.25)	0.50

Note: Bold values indicate statistical significance (p < 0.05). Abbreviations: BMI, body mass index; CRP, C-reactive protein; NSE, neuron-specific enolase; Glu, glucose; Plt, platelet; Hcy, homocysteine

**Fig 2:**
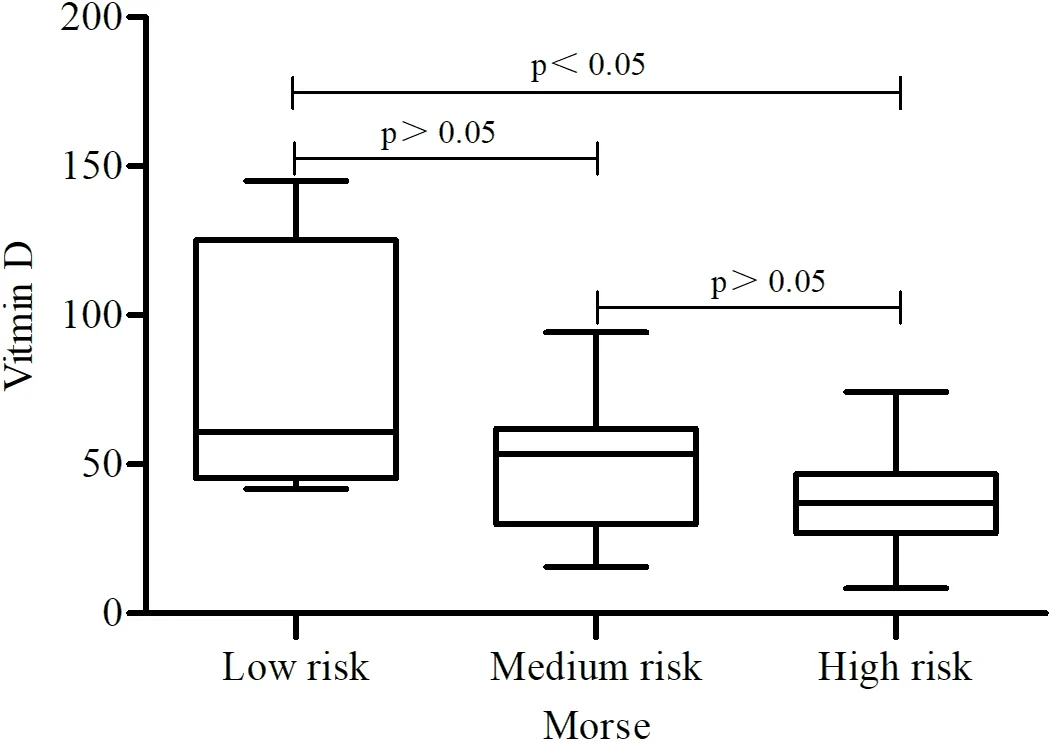
Distribution of serum 25(OH)D across fall‑risk subgroups. Serum 25‑hydroxyvitamin D concentrations (nmol/L) among participants stratified by low, moderate and high Morse Fall Scale fall risk groups.

Fig 2 shows the distribution of serum 25(OH)D concentrations across the low-, moderate-, and high-risk groups (all values in nmol/L). Serum 25(OH)D concentration was significantly lower in the high-risk group than in the low-risk group (p < 0.05).

Vitamin D status was categorized as sufficient (25[OH]D ≥ 50 nmol/L), insufficient (25 to <50 nmol/L), or deficient (<25 nmol/L). The distribution of vitamin D status differed significantly across the low-, moderate-, and high-risk groups (p < 0.001; Table 2).

**Table 2:** The vitamin D status of patients in each fall risk group.

Variables	*Morse*			*P*-value
	Low risk (n=4)	Medium risk (n=15)	High risk (n=112)	
Vitamin D status				**<0.001**
Deficient	1	6	91	
Insufficient	2	7	21	
Sufficient	1	2	0	

Multivariable binary logistic regression showed that higher serum 25(OH)D concentration was associated with lower odds of high fall risk (adjusted OR = 0.93 per 1-nmol/L increase, 95% CI 0.89–0.96; p < 0.001), whereas older age was associated with higher odds (adjusted OR = 1.07 per year, 95% CI 1.02–1.13; p = 0.01). Sex (p = 0.79) and BMI (p = 0.65) were not independently associated with high fall risk (Table 3).

**Table 3:** Multivariable logistic regression analysis of factors associated with high fall risk.

Variables	OR (95% CI)	*P*-value
Vitamin D	0.93 (0.89-0.96)	**<0.001**
Age	1.07 (1.02-1.13)	**0.01**
Sex	0.81 (0.18-3.75)	0.79
BMI	0.96 (0.79-1.16)	0.65

## Discussion

In this cross-sectional study of hospitalized patients, serum 25(OH)D concentrations were lowest in the high MFS-risk group, and vitamin D status differed across fall-risk categories. After adjustment for age, sex, and BMI, higher serum 25(OH)D concentration remained associated with lower odds of high fall risk, whereas older age was associated with higher odds. Although the unadjusted distribution of sex differed across the three MFS groups, sex was not independently associated with high fall risk in the multivariable model. These findings therefore do not support a conclusion that women had a higher independent risk than men.

Of all the reasons for injuries and deaths in older adults, falling tops the list. In 2014, falls contributed to the deaths of around 27,000 older adults. Also, 2.8 million were treated at EDs for non-fatal fall-related injuries. Furthermore, around 800,000 older adults were hospitalized due to falls [16,17]. As people get older, they tend to refrain from doing daily activities because they are scared of falling. The drop in outdoor activities and social activities with family and friends tend to reduce control with feelings, decreased social participation, and increase feelings of loneliness, anxiety and depressive symptoms [18]. Most falls are associated with multiple risk factors, such as frailty, unsteady gait, cognitive impairment and certain medicines. Addressing these risk factors can reduce falls significantly, research suggests [19].

Vitamin D is commonly used in osteoporosis management to support skeletal health. Some randomized trials and meta-analyses have reported benefits for muscle function, balance, or fall-related outcomes [20–24], whereas others have reported little or no benefit for fall prevention [25, 26], and improvements in strength do not necessarily translate into fewer falls [7, 27]. Emerging evidence suggests that supplementation effects may be context dependent. A recent systematic review and meta-analysis reported improvements in selected rehabilitation outcomes after stroke, although the evidence base was small [28]. In another clinical context, pooled randomized trials suggested improved diabetic foot-ulcer outcomes with vitamin D supplementation [29]. Neither study directly establishes fall prevention; rather, they illustrate that benefits may vary by baseline deficiency, disease state, outcome, dose, and co-interventions.

Our findings show an inverse association between serum 25(OH)D concentration and high MFS-defined fall risk, consistent with previous observational work [30]. However, several limitations should be considered. First, the sample was small and 112 of 131 participants were classified as high risk; this imbalance limited precision and may have introduced instability in intergroup and multivariable estimates. Second, the cross-sectional design precludes assessment of temporality or incident falls and does not support causal inference. Reverse causality is plausible because frailty or impaired mobility may reduce outdoor activity and ultraviolet-B exposure, thereby lowering vitamin D concentrations. Third, information on baseline mobility, previous vitamin D supplementation, season of blood sampling, medication use, and other potential confounders was unavailable or incomplete. Fourth, fall risk was assessed only with the MFS and was not corroborated using prospective fall surveillance or additional balance and mobility measures. Larger prospective cohorts and adequately powered intervention studies in vitamin-D-deficient populations are needed.

## Conclusion

In this hospitalized cross-sectional sample, lower serum 25(OH)D concentrations and older age were independently associated with high MFS-defined fall risk. Sex was not independently associated with high fall risk. Because the study cannot establish causality, prospective studies are needed to determine whether vitamin D deficiency precedes falls and whether targeted correction reduces clinically observed fall events.
